# The Role of Education, Monitoring, and Symptom Perception in Internet-Based Self-management Among Adolescents With Asthma: Secondary Analysis of a Randomized Controlled Trial

**DOI:** 10.2196/17959

**Published:** 2021-12-07

**Authors:** Thijs Beerthuizen, E R V M Rikkers-Mutsaerts, Jiska B Snoeck-Stroband, Jacob K Sont

**Affiliations:** 1 Department of Biomedical Data Sciences Medical Decision Making Leiden University Medical Center Leiden Netherlands; 2 Department of Pediatrics Leiden University Medical Center Leiden Netherlands

**Keywords:** web-based monitoring, internet self-management, adolescents, asthma, education, perception

## Abstract

**Background:**

Internet-based self-management programs improve asthma control and the asthma-related quality of life in adults and adolescents. The components of self-management programs include education and the web-based self-monitoring of symptoms; the latter requires adequate perception in order to timely adjust lifestyle or medication or to contact a care provider.

**Objective:**

We aimed to test the hypothesis that adherence to education and web-based monitoring and adequate symptom perception are important determinants for the improvement of asthma control in self-management programs.

**Methods:**

We conducted a subgroup analysis of the intervention group of a randomized controlled trial, which included adolescents who participated in the internet-based self-management arm. We assessed the impacts that attendance in education sessions, the frequency of web-based monitoring, and the level of perception had on changes in asthma control (Asthma Control Questionnaire [ACQ]) and asthma-related quality of life (Pediatric Asthma Quality of Life Questionnaire) from baseline to 12 months after intervention.

**Results:**

Adolescents who attended education sessions had significant and clinically relevant improvements in asthma control (ACQ score difference: −0.6; *P*=.03) and exhibited a nonsignificant trend of improvement in asthma-related quality of life (Pediatric Asthma Quality of Life Questionnaire score difference: −0.45; *P*=.15) when compared to those who did not adhere to education. Frequent monitoring alone did not improve asthma control (*P*=.07) and quality of life (*P*=.44) significantly, but its combination with education did result in improved ACQ scores (difference: −0.88; *P*=.02). There were no significant differences in outcomes between normoperceivers and hypoperceivers.

**Conclusions:**

Education, especially in combination with frequent web-based monitoring, is an important determinant for the 1-year outcomes of asthma control in internet-based self-management programs for adolescents with partly controlled and uncontrolled asthma; however, we could not establish the effect of symptom perception. This study provides important knowledge on the effects of asthma education and monitoring in daily life.

## Introduction

Asthma control is the goal in long-term asthma management, but despite the availability of effective therapies, this goal is not reached in three-quarters of patients with persistent asthma [1-3]. Adolescents form a vulnerable subgroup of patients with asthma that is characterized by a high prevalence of poor outcomes and high rates of morbidity and mortality. A lack of knowledge and perception of symptoms, especially when combined with a desire for independence and high-risk behaviors, interferes with adherence to asthma medication [4-6].

Asthma control and asthma-related quality of life can be improved in adults and adolescents, and the number of outpatient visits can be reduced by participating in an internet-based self-management (IBSM) support program [7-10]. In a previous randomized controlled trial, we assessed whether IBSM improved asthma control, asthma-related quality of life, and lung function in adolescents with partially controlled and uncontrolled asthma [11]. Adolescents allocated to the IBSM group of the trial showed improved asthma-related quality of life and asthma control within 3 months. However, these effects were not sustained during a longer period of time in a part of the intervention group. In the original paper, we did not assess which factors predicted favorable outcomes among the intervention group after the 12-month follow-up. These adolescents had access to education and self-monitored their asthma control, which are important components of self-management [7,11,12]. Adequately self-monitoring asthma control perceptions of airway obstruction symptoms seems crucial. Therefore, adherence to self-monitoring and education and the perception of airway obstruction might be important determinants of long-term outcomes in asthma self-management. This study is a secondary analysis of the Self-Management in Asthma Supported by Hospitals, Internet, Nurses and General Practitioners (SMASHING) trial [11], which we conducted in order to assess whether (1) adherence to education, (2) the amount of symptom monitoring, and (3) the level of symptom perception are related to improvements in asthma control and asthma-related quality of life in adolescents with partly controlled and uncontrolled asthma. We hypothesized that adherence to education sessions, frequent web-based monitoring, and an adequate perception of dyspnea are prerequisites to improving asthma control, asthma-related quality of life, and lung function after 12 months.

## Methods

### Patients

A detailed description of the methodology and patient recruitment process has been published before [11]. In short, adolescents aged between 12 and 18 years with a doctor’s diagnosis of persistent asthma were recruited from 35 general practices and the pediatric departments of 8 hospitals throughout the Netherlands. Patients requiring oral steroids for maintenance or patients with relevant comorbidities were excluded [11]. Only patients with partly controlled and uncontrolled asthma, as determined by having an Asthma Control Questionnaire (ACQ) score of >0.75 or an Asthma Therapy Assessment Questionnaire score of >1.0, were enrolled in the trial [13,14]. Patients were randomized via block randomization by a study coordinator who had no contact with the participants. After randomization, the baseline characteristics of the participants in the intervention arm and the control arm were similar [11]. In total, 11 of the 46 participants in the intervention group and 4 of the 44 participants in the usual care group dropped out. Furthermore, 9 of the remaining participants in the intervention group did not report secondary outcome measure (asthma control) results at 12 months after intervention [11].

### Design

To assess possible predictors of favorable outcomes in an IBSM support program, this study conducted an analysis of adolescents who participated in the intervention group of a randomized parallel trial (the SMASHING trial), which had a 1-year follow-up with 2-week evaluation periods at baseline and at 12 months [11]. In addition to usual care, adolescents in the IBSM intervention group received protocolized education in sessions that only involved small groups of participants. Furthermore, participants were asked to monitor their asthma control by using the ACQ weekly, and they received instant therapeutic advice according to a personal web-based treatment plan [11,13]. Participants could always report their daily symptoms and lung function by using a diary card (via the internet or short text messages) or by contacting the asthma nurse by phone or via the web. Apart from web-based information and interactive communication with the asthma nurse, education consisted of 2 asthma self-management education group sessions that were conducted within 6 weeks before participants entered the trial. Patient-tailored information about asthma self-management was provided in response to participants’ questions and individual concerns. Patients were asked to record asthma control outcomes by filling out the 7-item ACQ weekly. These included lung function (forced expiratory volume in 1 second [FEV_1_]), which was measured with a handheld electronic spirometer (Piko-1; nSpire Health Inc) and recorded in a personal page on a secure web application. They received instant feedback (based on a specific algorithm) on their levels of asthma control, including advice on how to adjust their medication according to a predefined personal treatment plan. At 0, 3, and 12 months, all participants monitored symptoms and lung function daily for 2 weeks, filled out the ACQ twice during those 2 weeks, and completed the Pediatric Asthma Quality of Life Questionnaire (PAQLQ) [15-18] once. To assess levels of symptom perception, participants were asked to visit the lung function laboratory to perform a bronchial challenge inhalation test involving methacholine at 12 months after intervention. If this visit could not be planned within 8 weeks from the 12-month evaluation period, the participants monitored symptoms, lung function, and ACQ entries for an additional 2 weeks before the methacholine challenge test. The studied group consisted of the patients in the intervention arm of the SMASHING study. Monitoring and education were only accessible to the intervention arm; hence, there are no such data for the participants in the control arm of the study.

### Measurements

#### Adherence to Education

Patients were defined as being adherent to education if they attended at least 1 of the 2 education sessions and as being nonadherent if they did not follow any education session.

#### Adherence to Monitoring

Adherence to monitoring was based on the frequency of monitoring ACQ entries during the follow-up period. Adolescents were asked to monitor ACQ entries weekly. We presumed that at the start of the trial, all participants would be motivated to perform monitoring, whereas during the follow-up of the program, only dedicated participants would continue to perform monitoring. We assumed that a monitoring frequency of at least 30 records in 12 months (full compliance in the first month and 50% compliance in the remaining period) would reflect adequate adherence to the intervention. Therefore, participants were divided into subgroups based on whether they adhered to ACQ monitoring (adherent subgroup: ≥30 ACQ entries; nonadherent subgroup: <30 ACQ entries).

#### Perception of Dyspnea

Perceptions of dyspnea were assessed in 2 ways, and patients were categorized as normoperceivers or hypoperceivers of dyspnea. First, perceptions of dyspnea were assessed during the methacholine inhalation challenge test. Methacholine was administered in doubling concentrations (range 0.15-640 μmol/mL). The challenge test was discontinued if the FEV_1_ decreased by more than 20% of the baseline value. All subjects were asked to assess the severity of their breathlessness before the first measurement of lung function, after the inhalation of a placebo (saline), and after receiving each incremental dose of methacholine. Patients rated the severity of the breathlessness that they experienced during the challenge test on a revised Borg scale [19]. The Borg scale is a category scale with ratio properties in which words describing increasing degrees of breathlessness are anchored to numbers ranging between 0 and 10, with 10 indicating the most severe degree of breathlessness. Perceptions of dyspnea were analyzed by using individual plots (Borg scores vs the percentage fall in FEV_1_) and expressed as slopes of the regression line (Borg slope). Based on the median of the Borg slope, patients were categorized as normoperceivers (≥median) or hypoperceivers (<median). Second, because the Borg slope was assessed at 12 months after intervention, to gain a longitudinal impression of perception, we also assessed a symptom slope by plotting the slope of the individual regression lines of daily symptom scores against the prebronchodilator-predicted FEV_1_ percentages during the follow-up. Based on the symptom slope, 2 independent observers (JKS and TB) categorized the adolescents as normoperceivers, hypoperceivers, hyperperceivers, or undefinable participants. Discordance was settled by consensus. Interobserver agreement was estimated by using the Cohen κ.

### Outcomes

The outcome parameters consisted of the difference between the baseline and 1-year outcomes of the PAQLQ and the individual averages of ACQ scores and FEV_1_ measurements from the 2-week diary cards. The minimal important change for both PAQLQ scores and ACQ scores was a difference of 0.5 points on their respective scales [20,21].

### Statistical Analysis

To assess the effect that education has on outcomes, we compared improvements in asthma-related quality of life and asthma control among adherent participants who had followed at least 1 of the 2 education sessions to those improvements in participants who did not follow any education session (the nonadherent participants), by using the Student 2-tailed *t* test.

We assessed whether adolescents who performed frequent monitoring (≥30 entries) clinically improved at 12 months after intervention in terms of asthma control (∆ACQ score≤−0.5) or quality of life (∆PAQLQ score≥0.5) by using the Student *t* test. We constructed a linear effects model to assess asthma control, quality of life, and lung function for the following three participant categories: no adherence to education and monitoring, only adherence to education, and adherence to both education and monitoring.

We also assessed whether normoperceivers clinically improved (ie, in terms of asthma control [∆ACQ score≤−0.5] or quality of life [∆PAQLQ score≥0.5]) more than hypoperceivers at 12 months after intervention by using the Student *t* test.

All analyses were performed with the Stata 11.0 (StataCorp LLC) statistical software package.

## Results

### Summary of Patient Characteristics

**Figure 1:**
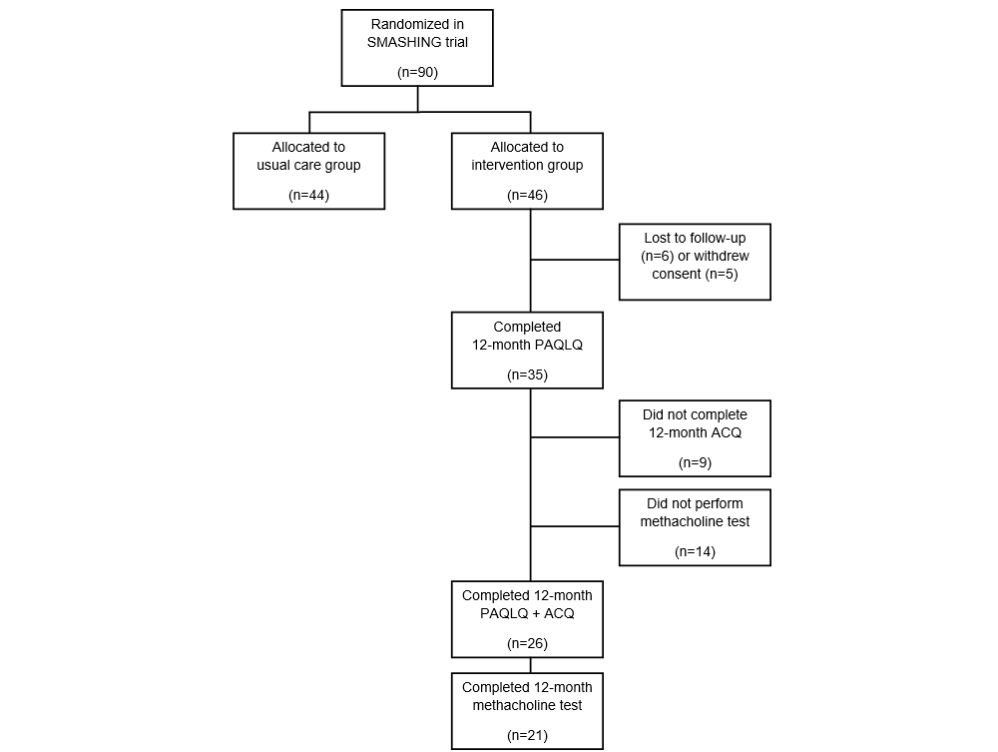
Study flow diagram. ACQ: Asthma Control Questionnaire; PAQLQ: Pediatric Asthma Quality of Life Questionnaire; SMASHING: Self-Management in Asthma Supported by Hospitals, Internet, Nurses and General Practitioners.

In the SMASHING study, 46 patients were randomized to the intervention arm. Of these participants, 11 dropped out during follow-up. Of the remaining 35 participants, 9 did not submit the final 12-month questionnaire. In an attempt to obtain at least the primary outcomes of the original study, we asked participants to fill out the PAQLQ. Hence, only 9 participants submitted this PAQLQ at the 12-month follow-up (Figure 1). The patient characteristics of the 35 adolescents in the IBSM group who completed the PAQLQ are presented in Table 1.

**Table 1:** Patient characteristics.

Characteristics		Internet-based self-management group (SMASHING^a^ study; n=35)	Nonadherence to education (n=13)	Adherence to education^b^ (n=22)
Males, n (%)		14 (40)	6 (46)	9 (36)
Age (years), mean (range)		14.1 (12-17)	13.7 (12-16)	14.1 (12-17)
**Care provider, n (%)**				
	General practitioner	12 (34)	2 (15)	10 (45)
	Pediatrician	23 (66)	11 (85)	12 (55)
FEV_1_^c^ (L), mean (range)		2.86 (1.74-4.31)	3.08 (1.99-4.30)	2.74 (1.74-4.26)
FEV_1_ (prebronchodilator; %), mean (range)		93 (65-125)	93.6 (73.2-117.7)	91.8 (64.5-125.9)
Daily inhaled corticosteroid dose (μg), mean (range)		353 (0-1000)	335 (100-1000)	402 (0-1000)
Pediatric Asthma Quality of Life Questionnaire score, mean (range)		5.78 (3.51-6.97)	5.84 (4.47-6.63)	5.75 (3.51-6.97)
Asthma Control Questionnaire score, mean (range)		1.22 (0.22-2.91)	1.03 (0.22-2.30)	1.33 (0.29-2.91)

^a^SMASHING: Self-Management in Asthma Supported by Hospitals, Internet, Nurses and General Practitioners.

^b^Adherence is defined as having attended at least 1 of the 2 education sessions.

^c^FEV_1_: forced expiratory volume in 1 second.

### Education

Of the 35 participants, 22 (63%) followed at least 1 education session (Table 1). Adolescents who were adherent to education showed significant improvements between 0 and 12 months in terms of asthma control (∆ACQ score: mean −0.60; 95% CI −1.12 to −0.08; *P=*.03) when compared to those who were not adherent to education (Table 2). This difference was clinically relevant. No statistically significant difference was found for asthma-related quality of life (∆PAQLQ score: mean 0.45; 95% CI −0.17 to 1.07; *P=*.15) between these two groups (Table 3).

**Table 2:** Asthma control improvement dichotomized by education, monitoring, and perception. A lower (negative) score represents a more favorable outcome.

Categories			ACQ6^a^ score (n=26), mean (95% CI)	*P* value
**Education**				
	Nonadherence (n=7)		−0.015 (−0.68 to 0.65)	N/A^b^
	Adherence (n=19)		−0.62 (−0.86 to −0.37)	N/A
	Difference		−0.60 (−1.12 to −0.08)	.03
**Monitoring**				
	<30 entries (n=16)		−0.28 (−0.56 to 0)	N/A
	≥30 entries (n=10)		−0.73 (−1.23 to −0.24)	N/A
	Difference		−0.45 (0.94 to 0.05)	.07
**Education and monitoring**				
	**Comparison 1**			
		Education nonadherence and <30 monitoring entries (n=5)	−0.05 (−0.92 to 0.82)	N/A
		Education adherence and ≥30 monitoring entries (n=8)	−0.93 (−1.32 to −0.53)	N/A
		Difference	−0.88 (−1.59 to −0.17)	.02
	**Comparison 2**			
		Education adherence and <30 monitoring entries (n=11)	−0.39 (−0.67 to −0.11)	N/A
		Education adherence and ≥30 monitoring entries (n=8)	−0.93 (−1.33 to −0.53)	N/A
		Difference	−0.54 (−0.98 to −0.11)	.02
**Borg score**				
	Hypoperceiver (n=8)		−0.18 (−0.72 to 0.36)	N/A
	Normoperceiver (n=6)		−0.66 (−1.25 to −0.07)	N/A
	Difference		−0.48 (−1.20 to 0.24)	.17
**Symptom slope**				
	Hypoperceiver (n=15)		−0.49 (−0.91 to −0.07)	N/A
	Normoperceiver (n=7)		−0.49 (−0.86 to −0.13)	N/A
	Difference		0 (−0.26 to 0.26)	.99

^a^ACQ6: 6-item Asthma Control Questionnaire.

^b^N/A: not applicable.

**Table 3:** Asthma-related quality of life improvement dichotomized by education, monitoring, and perception. A higher (positive) score represents a more favorable outcome.

Categories			PAQLQ^a^ score (n=35), mean (95% CI)	*P* value
**Education**				
	Nonadherence (n=13)		−0.094 (−0.65 to 0.47)	N/A^b^
	Adherence (n=22)		0.36 (−0.01 to 0.73)	N/A
	Difference		0.45 (−0.17 to 1.07)	.15
**Monitoring**				
	<30 entries (n=24)		0.11 (−0.20 to 0.42)	N/A
	≥30 entries (n=11)		0.36 (−0.42 to 1.15)	N/A
	Difference		0.25 (0.41 to 0.91)	.44
**Education and monitoring**				
	**Comparison 1**			
		Education nonadherence and <30 monitoring entries (n=11)	0.07 (−0.42 to 0.55)	N/A
		Education adherence and ≥30 monitoring entries (n=9)	0.66 (0.01 to 1.32)	N/A
		Difference	0.60 (−0.15 to 1.34)	.11
	**Comparison 2**			
		Education adherence and <30 monitoring entries (n=13)	0.14 (−0.32 to 0.61)	N/A
		Education adherence and ≥30 monitoring entries (n=9)	0.66 (0.01 to 1.32)	N/A
		Difference	0.52 (−0.21 to 1.25)	.15
**Borg score**				
	Hypoperceiver (n=8)		−0.02 (−0.60 to 0.57)	N/A
	Normoperceiver (n=10)		0.09 (−0.46 to 0.63)	N/A
	Difference		0.10 (−0.64 to 0.84)	.77
**Symptom slope**				
	Hypoperceiver (n=16)		0.25 (−0.33 to 0.83)	N/A
	Normoperceiver (n=7)		0.17 (−0.39 to 0.74)	N/A
	Difference		0.079 (−0.84 to 1.00)	.86

^a^PAQLQ: Pediatric Asthma Quality of Life Questionnaire.

^b^N/A: not applicable.

### Monitoring of Asthma Control

We found no statistically significant difference in improvements in ACQ scores between adolescents who had more than 30 monitoring entries compared to those who conducted monitoring less frequently (∆ACQ score: mean −0.45; 95% CI −0.94 to 0.045; *P*=.07) or in improvements in asthma-related quality of life (∆PAQLQ score: mean 0.25; −0.41 to 0.91; *P*=.44; Table 2 and 3). However, in adolescents who were adherent to both education and the frequent monitoring of ACQ entries (≥30 entries), there was a significant and clinically relevant improvement in asthma control (∆ACQ score: mean −0.88; 95% CI −1.59 to −0.17; *P*=.02) when compared to such improvements in adolescents who were not adherent to education and conducted monitoring less frequently (Table 2). The group of patients who were adherent to both education and monitoring also showed better asthma control compared to that of adolescents who adhered to education but had less than 30 monitoring entries (∆ACQ score: mean −0.54; 95% CI −0.98 to −0.11; *P*=.02; Table 2). The same trend was found for the difference in PAQLQ scores, but this did not reach significance, as shown in Table 3 (*P*=.15)*.* A linear effects model for assessing the impacts of no adherence, only education, and adherence to both education and monitoring showed that adherence to education and frequent monitoring had a favorable effect on asthma control (ACQ score: mean −0.45; 95% CI −0.74 to −0.16; *P*=.004). However, their effects on quality of life (PAQLQ score: mean 0.29; 95% CI −0.07 to −0.64; *P*=.11) and lung function (FEV_1_ score: mean 0.08; 95% CI −0.16 to 0.33; *P*=.49) were not significant (Table 4).

**Table 4:** Lung function improvement dichotomized by education, monitoring, and perception. A higher (positive) value represents a more favorable outcome.

Categories			FEV_1_^a^ value (n=29), mean (95% CI)	*P* value
**Education**				
	Nonadherence (n=9)		0.12 (−0.10 to 0.33)	N/A^b^
	Adherence (n=20)		0.31 (0.061 to 0.56)	N/A
	Difference		0.19 (−0.41 to 0.58)	.32
**Monitoring**				
	<30 entries (n=18)		0.24 (−0.04 to 0.52)	N/A
	≥30 entries (n=11)		0.27 (0.01 to 0.44)	N/A
	Difference		0.03 (−0.40 to 0.35)	.89
**Education and monitoring**				
	**Comparison 1**			
		Education nonadherence and <30 monitoring entries (n=7)	0.16 (−0.08 to 0.39)	N/A
		Education adherence and ≥30 monitoring entries (n=9)	0.33 (0.19 to 0.46)	N/A
		Difference	0.17 (−0.06 to 0.40)	.13
	**Comparison 2**			
		Education adherence and <30 monitoring entries (n=11)	0.29 (−0.18 to 0.77)	N/A
		Education adherence and ≥30 monitoring entries (n=9)	0.33 (0.19 to 0.46)	N/A
		Difference	0.04 (−0.47 to 0.55)	.88
**Borg score**				
	Hypoperceiver (n=8)		0.44 (−0.13 to 1.00)	N/A
	Normoperceiver (n=8)		0.12 (−0.20 to 0.43)	N/A
	Difference		0.32 (−0.26 to 0.91)	.26
**Symptom slope**				
	Hypoperceiver (n=16)		0.19 (0.01 to 0.38)	N/A
	Normoperceiver (n=7)		0.10 (−0.18 to 0.39)	N/A
	Difference		0.09 (−0.23 to 0.41)	.57

^a^FEV_1_: forced expiratory volume in 1 second.

^b^N/A: not applicable.

### Perception

A total of 21 participants in the IBSM group performed the methacholine test and had Borg scores (Table 5). They were categorized as normoperceivers (n=11) and hypoperceivers (n=10). Based on the symptom slope, participants in the IBSM group were categorized as normoperceivers (n=17), hypoperceivers (n=10), hyperperceivers (n=1), and undefinable participants (n=18; interobserver agreement: κ=0.67). There was no strong relationship between the Borg slope and symptom slope (Spearman correlation coefficient [*R*_s_]: −0.29). There were no statistically significant differences in outcomes between normoperceivers and hypoperceivers based on the Borg slopes for asthma control (∆ACQ score: mean 0.48; *P*=.17) and asthma-related quality of life (∆PAQLQ score: mean −0.10; *P*=.77; Table 5). Similarly, no significant differences in outcomes were found if perception was based on the symptom slope (Table 5).

**Table 5:** Outcomes in normoperceivers and hypoperceivers based on Borg and symptom slopes.

Slopes and outcomes		Value (number of normoperceivers)	Value (number of hypoperceivers)	Difference (95% CI)	*P* value
**Borg slope**					
	∆mACQ_0-12_^a^	−0.66 (6)	−0.18 (8)	0.48 (−0.24 to 1.2)	.17
	∆PAQLQ_0-12_^b^	0.09 (10)	0.02 (8)	−0.10 (−0.84 to 0.63)	.77
	∆mFEV_1_,_0-12_^c^	0.12 (8)	0.44 (8)	0.32 (−0.26 to 0.90)	.26
**Symptom slope**					
	∆mACQ_0-12_	−0.49 (15)	−0.49 (7)	0 (0.65 to 0.78)	>.99
	∆PAQLQ_0-12_	0.25 (16)	0.17 (7)	0.08 (−0.84 to 0.10)	.86
	∆mFEV_1_,_0-12_	0.19 (16)	0.10 (7)	0.09 (−0.23 to 0.41)	.57

^a^∆mACQ_0-12_: change in mean Asthma Control Questionnaire scores from 0 months to 12 months after intervention.

^b^∆PAQLQ_0-12_: change in mean Pediatric Asthma Quality of Life Questionnaire scores from 0 months to 12 months after intervention.

^c^∆mFEV_1,0-12_: change in mean forced expiratory volume in 1 second values from 0 to 12 months after intervention.

## Discussion

This study showed that participation in education sessions, especially in combination with frequent monitoring, is an important determinant for the 1-year outcomes of asthma control in IBSM programs for adolescents with partly controlled and uncontrolled asthma.

Attending at least 1 education session was a predictor of significant improvement in asthma control during the follow-up when compared to not attending any education session. Frequent monitoring alone was not a predictor of significant improvement in asthma control. However, for the group of education-adhering adolescents, frequent monitoring was a predictor of even further improved asthma control when compared to frequent monitoring in the nonadherent group. We did not observe important improvements in asthma-related quality of life in these groups. Differences in quality of life and asthma control were found between the subgroup that was nonadherent to both education and monitoring and the subgroup that was adherent to both education and monitoring. However, these subgroups were too small for establishing a solid conclusion. Our linear effects model showed the favorable effect that education and monitoring have on asthma control. No significant differences in asthma control or quality of life were observed between the small groups of normoperceivers and hypoperceivers, as determined by the Borg score (asthma control: *P*=.17; quality of life: *P*=.77) and by the constructed “real-life” symptom slope (asthma control: *P*=.99; quality of life: *P*=.86).

Although no causal relationship could be established due to the design of this study, the findings contribute to previous literature reporting that education and monitoring are generally associated with improved asthma control; however, results have been mixed for improvements in quality of life [22,23]. A recent study showed that thorough education, especially in peer groups, can have a sustainable beneficial effect [23]. Further, a large cohort study established that education should be an integral part of effective asthma treatment, as it can result in fewer asthma exacerbations [24]. Our study highlights both the importance and the challenge of adherence to asthma therapy in adolescents [25].

Several limitations need to be addressed. High dropout rates are a common challenge in studies with adolescent populations. Consequently, our small sample size could have contributed to a loss of statistical power and an increase in uncertainty for several outcomes. Nonetheless, several significant and clinically relevant predictors of improved asthma control were established in this study. Enrolling a higher percentage of the eligible population of 688 patients would have been desirable for increasing statistical power. We note that in the randomized controlled trial, monitoring was performed by using short text messages, and this was a more laborious process compared to other easy-to-use methods, such as using mobile phone apps, that can be implemented by using modern mobile communication technology. We believe that a simple web application and the absence of long questionnaires (eg, the questionnaires to which adolescents had to commit themselves in order to be enrolled in the trial) would help with increasing adolescent participation in self-management interventions in clinical practice.

With respect to possible selection bias, one could argue that the improvement in asthma control in patients who adhered to education and monitoring might not have been due to adherence to the intervention itself but, instead, might have been due to the selection of a cooperative and adherent patient population that can be expected to exhibit better health statuses. However, even within a potentially adherent patient group, we observed further improvements in asthma control among patients who attended education sessions.

Unfortunately, not all participants completed the methacholine inhalation challenge test. Therefore, we constructed a “real-life” measure for perceptions of symptom severity (ie, the symptom slope). Although we found good interobserver agreement for this novel measure, there were no important differences among comparison groups. Therefore, the absence of differences in symptom perceptions did not seem to depend on our chosen methodology or a lack of statistical power.

With regard to external validity, one could argue that only highly motivated adolescents participate in extensive studies such as ours. Therefore, our results might not apply to the entire population of adolescents with asthma. We however argue that the problems of adolescent chronic health care do not lend themselves well to a one-size-fits-all approach. Although we might not reach all adolescents, promoting health in motivated groups is desirable in itself, and effective self-management in motivated adolescents might increase motivation among youth. Therefore, we believe that our results provide useful insights for supporting self-management in adolescents with asthma.

Our results imply that following at least 1 educational group session results in a significant and clinically relevant improvement in asthma control when compared to following no education at all. This emphasizes and supports the importance of educating adolescents with asthma, which is in line with several other studies [22,26,27]. Our results show that adolescents who follow education and conduct frequent monitoring during a study exhibit significantly better and clinically relevant changes in asthma control after 12 months. The same trend was seen with regard to asthma-related quality of life, but this trend was not statistically significant (*P*=.15). Therefore, in adolescents with asthma who follow an IBSM program, both education and monitoring seem to be important factors in achieving better asthma control and asthma-related quality of life.

In our study, we could not find a significant difference in the results of adolescents who were normoperceivers and those who were hypoperceivers. It can be argued that the assessment of the perception of airway obstruction during a methacholine challenge does not reflect real-life symptom perception. However, this perception, which was assessed based on the relationship between symptoms and lung function, was not related to improvements in asthma control and quality of life. This suggests that the role of symptom perception in self-management is complex, and this illustrates that the concept of perception is difficult to capture with indices based on the relationship between symptom scores and lung function.

We conclude that the results of our study emphasize the importance of education adherence and frequent monitoring in improving asthma control among adolescents with partly controlled and uncontrolled asthma. No significant association between improvements in asthma control and perceptions of asthma control was found.

## Abbreviations

ACQ: Asthma Control Questionnaire
FEV_1_: forced expiratory volume in 1 second
IBSM: internet-based self-management
PAQLQ: Pediatric Asthma Quality of Life Questionnaire
SMASHING: Self-Management in Asthma Supported by Hospitals, Internet, Nurses and General Practitioners
